# West Ham, Stratford, and South Essex Hospital

**Published:** 1894-02-17

**Authors:** 


### The Institutional Workshop.

### WITHIN THE HOSPITALS.

### WEST HAM, STRATFORD, AND SOUTH ESSEX HOSPITAL.

(By Our Special Commissioner.)

We visited this hospital with considerable interest, because its establishment signalised a new departure in hospital matters in London. The rapid growth of the population, and a proportionate increase of cases of accident and sickness, impelled the inhabitants, who are mainly of the artisan class, to combine with the object of erecting and maintaining a hospital for their accommodation. Heretofore the inhabitants had depended mainly on the London Hospital, and it is pleasant to be able to note that this movement received the benevolent encouragement of the older institution, and that H.R.H. the Duke of Cambridge, its President, laid the foundation-stone on June 18th, 1888. The total cost was £7,000, the whole of which was raised in the district, the lady residents in which collected a further sum of £1,065 towards furnishing the hospital. We mention these facts to show, that so far as the people of West Ham were concerned, everything was done to produce a complete hospital, worthy of the public spirit and liberality which was so encouragingly manifested. Ever since 1861 a dispensary had been attending to out and home patients in the district, and as it was found impossible to develop this into a hospital the new hospital was erected in close proximity to the dispensary.

#### The Present Hospital Buildings.

We wish particularly to direct our readers' attention to the fact that this new hospital was opened so recently as April 23rd, 1890, that is, less than four years ago. This circumstance, and the fact that it is situated within a comparatively short distance of the centre of London, led us to hope that we should find a building replete with everything which modern construction and sanitation render possible. To our surprise we found a building which had evidently been designed without adequate knowledge of the requirements of a modern hospital, and which contained practically no accommodation suitable for the nursing staff; that the Matron's rooms were so constructed and placed as to be calculated to deprive her of health and sleep; that the two main wards, though adequately proportioned, were sadly deficient in the usual offices and lavatories; that the fittings were unsuited, both in size and character, to hospital purposes; and that generally a great opportunity for building a hospital worthy of modern science had been lost. So much struck were we with these facts that we would invite the attention of all who are interested in hospital contruction to pay an early visit to West Ham in order to learn a practical lesson of what to avoid.

We have no wish to attempt to apportion the responsibility for this failure, but as the institution, owing to the increase in the number of patients, is about to be extended, we would earnestly impress upon the committee the importance of summoning to their aid a skilled expert in hospital matters to be associated with their architects, and so to provide that further mistakes shall be avoided as far as practicable. It requires no special knowledge to realise, that bad as the sleeping accommodation for the nurses is at the present time, several of the rooms will be made practically unfit for occupation if a large building is to be erected within a few feet of the only windows which they at present contain. In our view, not one single stone of the new building should be erected until a careful plan of the existing buildings has been prepared, together with a plan of the proposed extensions, and a report has been invited from an independent authority, who has a sound knowledge of hospitals and their requirements. If any other course be adopted, we shall probably find, that in a few years time, the new hospital buildings may have to be pulled down or vacated, because the committee may find it unjustifiable to continue to treat patients therein.

Briefly, we may state that the hospital as it at present stands, judged by a high standard, contains no proper accommodation for the staff. Two nurses are placed in a room which would be uncomfortable for one occupant. The Matron's rooms are dark, and her bed-room is next to the isolation ward, where noisy patients are placed, whilst the only window is above the laundry, and so it is impossible to open it. The floors of the wards afford startling evidence of the want of knowledge and experience which has dogged the footsteps of the committee in their endeavours to secure an efficient hospital. Someone appears to have been instructed, about six months ago, to polish these floors, with the result that they are at present in the very worst condition we have ever seen any floors anywhere. It is possible to take up with the fingers long splinters of wood, showing that the wrong material was used in the first place, whilst the present appearance of the floors is a grave disfigurement to the wards. The fittings in the sculleries are ridiculously small and ill-adapted to the purposes of a hospital, and the metal baths and other appliances in the lavatories ought never to have been used in a modern hospital. The ventilation everywhere is defective. The large wards are good, but that for women and children was overcrowded, and it is surprising how the boys' ward, having regard to its construction and insanitation, has found a place in a modern hospital. The pity of it is that this ward was erected by funds raised by teachers and scholars in the district, as it is in reality an object lesson of much to avoid in ward construction.

#### The Administration of the Hospital.

Judging by appearances, we should say that the present staff have done their utmost to make the best of the unsuitable buildings which they have to administer. The attempts at decoration, and the introduction of little adjuncts to the wards point to a careful endeavour to husband limited funds wisely and well. The kitchens, at the top of the building, form one of the best features in the hospital, though we noticed that the larders are inadequately ventilated, like other parts of the building. There are two resident medical officers, and only one bath is provided for the staff of both sexes.

The nursing is done by three sisters (two day and one night) and seven probationers. This staff is inadequate, and the sister of the women's and children's wards should certainly have the aid of a charge nurse. The casualities are very numerous, having exceeded 10,000 cases during the last year. The average daily attendance in the out-patient department is 200, and the dressing of these casualties is in charge of one probationer-nurse—a fact which should receive the immediate attention of the committee, with a view to the provision of further assistance without delay.

How to Improve the Hospital.

We hope that our criticisms, which were urgently called for, will be taken by the committee in good part. We incline to the belief that they are not to blame in the matter of the buildings, and the happy faces of the patients and the home-like aspects of the hospital prove that those responsible for the management take genuine interest in the well-being of the patients. They evidently do their utmost to make the patients comfortable, and to promote their speedy recovery. It was also pleasant to notice that the roof of the hospital has been converted into a skating-rink, where the nurses can get reasonable exercise in fine weather, and to hear that the passengers of the great ocean-going steamers are in the habit of sending their deck-chairs to the hospital, so that the roof may be made a pleasant resort in warm weather. Do not let the committee be discouraged by the condition of affairs as we found them, Let that condition rather stimulate them to renewed efforts in the direction of completeness and efficiency. Then, but not otherwise, it ought to be speedily possible, by reconstruction, to remove the most flagrant of the defects to be found in the existing buildings, and to secure that the new extensions shall not only provide adequate accommodation for more patients, but be so designed as to render the completed hospital adequate in all respects for the work it has to do. Accommodation must be found for the whole of the nursing staff, new quarters should be provided for the Matron, and possibly for one or both of the medical officers; care must be taken that the new buildings do not in any way interfere with the healthiness or lighting of the existing wards and offices, and, if necessary, an attempt must be made to purchase more land, so as to make the site capable of containing the new buildings and providing for further necessary extension. We think that the history of the establishment of this hospital, and the success which attended the combined efforts of the inhabitants to raise the required funds, may be taken to prove that the present managers are genuinely anxious to secure a hospital which shall be replete with every modern requisite. We are confident that this result may yet be accomplished, providing they will delay the erection of the new buildings until they have obtained the advice of a competent authority, who, in conjunction with the architects, should be charged with the responsibility of stating how the desired end can best be arrived at.

